# Cerebrospinal Fluid Leaks and Healthcare Costs Associated With Tethered Cord Release and Filum Terminale Sectioning

**DOI:** 10.7759/cureus.80102

**Published:** 2025-03-05

**Authors:** Andrea Shehaj, Alexandra DiGiovanni, David Millar, Yahya Khan, Arba Cecia, Hannah E Wilding, Mallory Peterson, Elias B Rizk

**Affiliations:** 1 Department of Neurosurgery, Penn State College of Medicine, Hershey, USA; 2 Department of Medicine, Loyola University Chicago Stritch School of Medicine, Maywood, USA; 3 Department of Neurosurgery, Penn State Health Milton S. Hershey Medical Center, Hershey, USA

**Keywords:** csf leak, filum terminale sectioning, healthcare costs, tethered cord, tethered cord release

## Abstract

Introduction: Tethered cord syndrome is a condition caused by anatomical restrictions that limit the normal movement of the spinal cord, resulting in metabolic and vascular abnormalities that lead to hypoxia and jeopardize function. This study aims to comprehensively analyze the incidence of cerebrospinal fluid (CSF) leaks and the economic burden in pediatric patients who have undergone tethered cord release (TCR) and sectioning of filum terminale, stratified by the duration of postoperative recumbency.

Methods: After approval from the Institutional Review Board, a retrospective chart review of a tertiary medical center was performed. Patients were stratified into two cohorts based on the time spent in the recumbent position (cohort 1: zero days vs. cohort 2: greater than or equal to one day).

Results: Cohort 1 (n = 35) had a mean age of 9.14 ± 2.51 years, and cohort 2 (n = 25) had a mean age of 3.94 ± 0.87 years. The mean procedure duration was shorter in cohort 1 at 2.318 hours (1.22-4.63 hours) compared to 2.74 hours (2.20-3.97 hours) in cohort 2. CSF leaks were observed in one patient in cohort 1 (2.8%) and one patient in cohort 2 (4%), which was determined to be statistically insignificant through Fisher’s exact test (p > 0.99). Cohort 1 had a higher overall frequency of total postsurgical complications with six patients (17.1%) compared to three patients in cohort 2 (12%) (p = 0.72). The most common complication was postsurgical infections, which occurred in four patients in cohort 1 (11%) and one in cohort 2 (4%). Mean postoperative hospital stays were 1.969 days for cohort 1 and 1.68 days for cohort 2. The inpatient hospital costs accrued and calculated in 2024 U.S. dollars were $13,742 and $14,650 for cohorts 1 and 2, respectively (p = 0.79).

Conclusion: The increased burden and inpatient days needed to place patients in the supine position post-TCR are likely not justified in the setting of comparable CSF leak rates.

## Introduction

Tethered cord syndrome (TCS) is a clinical condition caused by anatomical restrictions that limit the normal movement of the spinal cord. This results in vascular abnormalities characterized by hypoxia that can jeopardize the function, development, and makeup of the spinal cord [1]. Tethering can be acquired (secondary) or congenital (primary). The resultant tension can cause a broad spectrum of symptoms, some of which include neurological deficits such as weakness or sensory abnormalities, gastrointestinal dysfunction, urinary incontinence, and musculoskeletal abnormalities [2,3]. TCS is associated with congenital spinal dysraphism, such as spina bifida, fatty filum terminale, split cord malformation, and dermal sinus tracts [4-6].

If left untreated, TCS can progressively worsen symptoms due to the ongoing stretch and strain on the spinal cord, particularly as a pediatric patient grows. For patients with new or worsening symptoms, surgical intervention is often required. Tethered cord release (TCR) is the typical treatment for simple tethered cord causes, such as a fatty filum. Typically, it involves a single-level lumbar laminectomy, intradural exploration, and the coagulation and cutting of the filum terminale [7]. TCR is a relatively safe procedure with low morbidity and mortality rates [8]. Despite the generally favorable outcomes, the postoperative care of TCR patients is still evolving, particularly regarding practices to minimize the risk of complications such as cerebrospinal fluid (CSF) leaks.

Postoperative care for TCR often involves keeping patients in the recumbent position for one to three days, a practice hypothesized to reduce the risk of CSF leaks [9-11]. However, limited studies have sufficiently investigated this hypothesis. Thus, investigating if postsurgical recumbent positioning is necessary for CSF leak prevention may be overlooked in the literature. The objective of this study is to comprehensively analyze the incidence of CSF leaks in pediatric patients who have undergone TCR, stratified by the duration of their postoperative recumbency. Additionally, this study aims to identify correlations between surgery length, postoperative care, and complication rates of TCR, with a specific focus on CSF leaks. Importantly, this study includes a cost analysis to determine the monetary burden on TCS patients. This study aims to provide insight into optimal postoperative care for pediatric patients with TCS and contribute to the growing literature on TCS.

## Materials and methods

Study design

**Figure 1:**
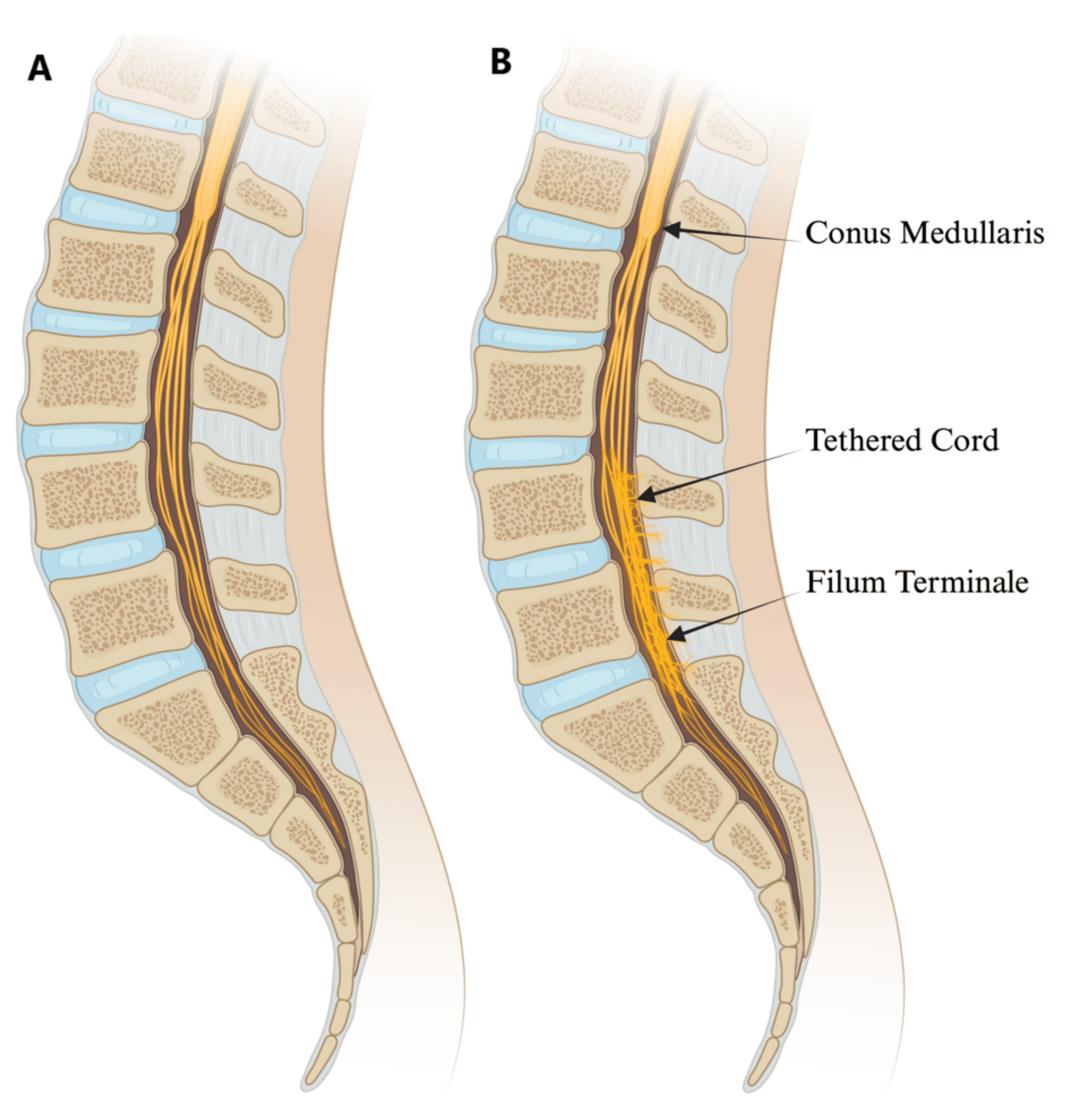
Illustration depicting (A) a normal spinal cord and (B) a tethered spinal cord Image credit: This is an original image created by the author Andrea Shehaj using BioRender.com

A retrospective analysis consisting of chart reviews of a tertiary medical center was performed. The Institutional Review Board approved this study before chart reviews. Patients in this study had undergone TCR and filum sectioning by various neurosurgeons at one institution who differed in their postoperative recumbency approach between January 2010 and December 2023. Patients were separated into two cohorts based on the duration spent in the recumbent position (zero days vs. greater than or equal to one day). Cohort 1 spent zero days in the recumbent position, and cohort 2 spent greater than or equal to one day in the recumbent position and were asked to remain flat until at least postoperative day 1. Given that the development of tethered spinal cord syndrome typically occurs due to congenital growth defects of the neural tube during fetal development, the cohorts consisted predominantly of pediatric patients (<18 years old), but older patients were not excluded from the analysis. Patients followed for less than six months after the surgical procedure were excluded from the study. A visual depiction of TCS is presented in Figure 1.

Study population and data recorded

**Figure 2:**
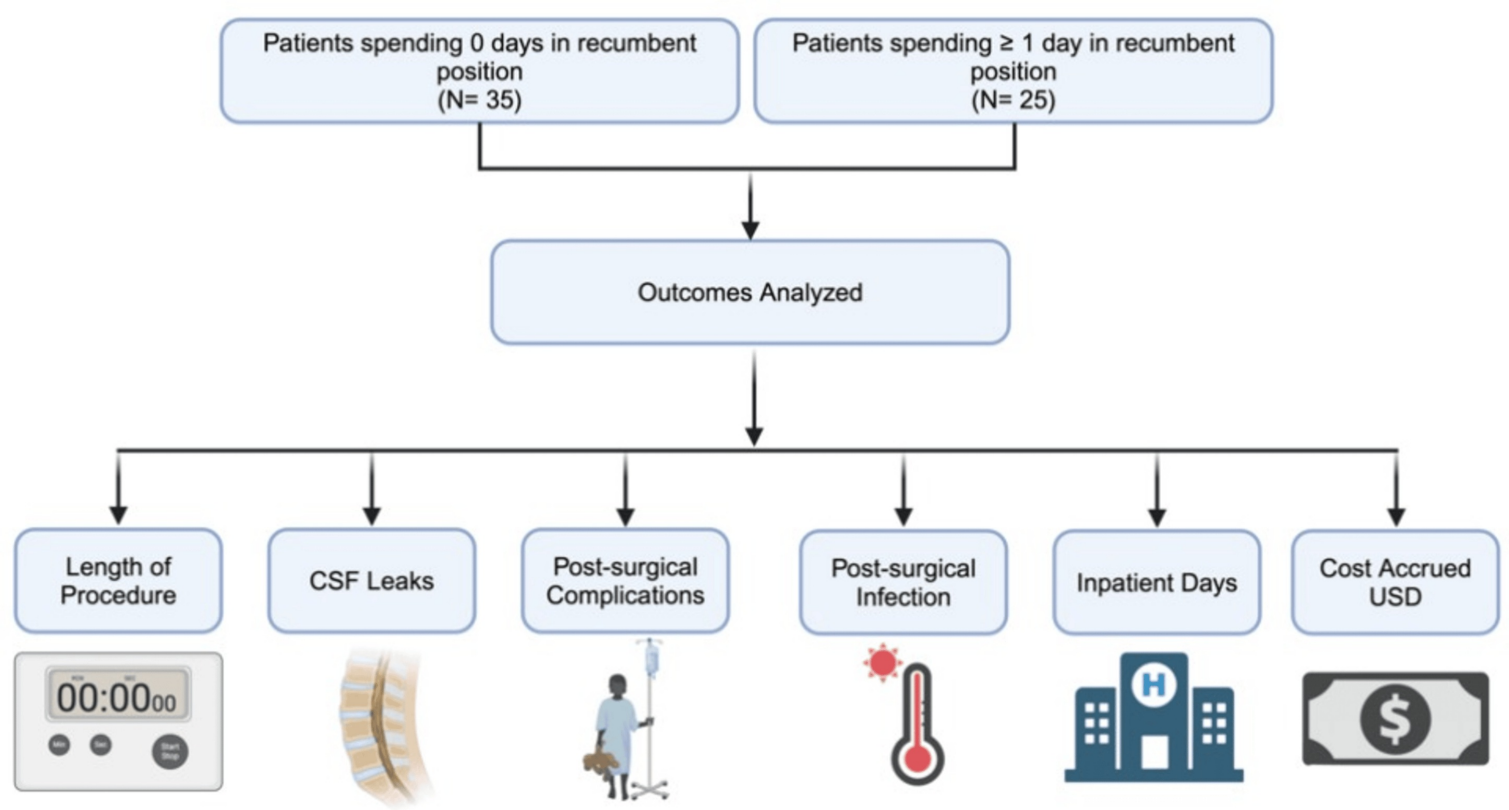
Study design and outcome variables analyzed Outcomes analyzed include length of procedure, CSF leak prevalence, postsurgical complications, postsurgical infections, inpatient days, and cost accrued in U.S. dollars CSF: cerebrospinal fluid Image credit: This is an original image created by the author Andrea Shehaj using BioRender.com

Upon Institutional Review Board approval, patients who had undergone TCR and filum sectioning were reviewed to investigate CSF leaks as the primary outcome following surgical intervention. Variables collected included date of birth, age during surgical intervention, sex, duration of TCR, CSF leaks, inpatient days, and cost calculations in U.S. dollars during the patient’s hospital stay. CSF leaks were determined by diagnostic imagining or the patient needing surgical intervention. Patients were also investigated for other surgically related complications, including postoperative infection. The study design and variables investigated during data collection are highlighted in Figure 2. The number of days patients spent in the recumbent position was not revealed to coauthors during the retrospective chart review. The reviewers were blinded to each provider's recumbency preference.

Data analysis

A Fisher's exact test was utilized to assess the association between instances of CSF leaks and days spent in the recumbent position (zero days vs. greater than or equal to one day). Fisher's exact test was also chosen since it does not rely on significant sample size assumptions due to the lower sample sizes presented in this study. In determining a relationship between the categorical variables, a p value of 0.05 was used as the standard for statistical significance when conducting a Fisher's exact test. The total cost for inpatient days was calculated throughout the year and adjusted in U.S. dollars. Prism GraphPad (Dotmatics, Boston, MA) was utilized for all data analysis and data representation.

## Results

Patient population demographics

The two patient cohorts compared in this study included patients who underwent TCR and had different postsurgical guidelines regarding recumbency during recovery. Cohort 1 included patients not required to be in a recumbent position after surgery, while cohort 2 included patients who spent at least one day in a recumbent position following surgery. Thirty-five patients in cohort 1 and 25 patients in cohort 2 were included for analysis. A relatively older patient population was noted in cohort 1, with a mean age ± standard error of the mean of 9.14 ± 2.51 years. The mean age in years of cohort 2 was calculated to be 3.94 ± 2.51 years. Cohort 1 comprised 14 male patients (40%), whereas cohort 2 comprised 11 male patients (46%). Table 1 further highlights these patient demographics.

**Table 1:** Demographics of patients in designated cohorts Values are reported for both cohorts 1 and 2, divided by the duration of postsurgical days spent in a recumbent position by patients who underwent TCR. Cohort 1 represents patients who spent zero days in the recumbent position, and cohort 2 represents patients who spent at least one day in the recumbent position. The sample size for each cohort, sex distribution, and average age ± standard error of the mean have been outlined TCR: tethered cord release

Variable of interest	Cohort 1 (zero days)	Cohort 2 (greater than or equal to one day)
Sample size (number of patients)	35	25
Age in years, mean ± standard error of the mean	9.14 ± 2.51	3.94 ± 2.51
Sex		
Male	14	11
Female	21	14

TCR outcomes

The average procedure length for cohort 1 was 2.3 hours, ranging from 1.22 to 4.63 hours. This was shorter than cohort 2 at 2.7 hours, ranging from 2.20 to 3.97 hours. Patients in cohort 1 had a lower incidence of CSF leaks, with one patient (2.8%) in cohort 1 and one patient (4%) in cohort 2 (p > 0.99). The former more frequently had postsurgical complications, with six patients (17.1%) in cohort 1 and three patients (12%) in cohort 2. Complications for those in cohort 1 included areflexia and a urinary leak in one patient (2.8%) with Guillain-Barre syndrome, which likely related to the development of these complications, difficulty voiding in one patient (2.8%), and local infection at the surgical site. Local infection was assessed as an independent variable, which occurred in four patients (11%) in cohort 1 and one patient (4%) in cohort 2. Other complications of patients in cohort 2 included fever in one patient (4%) and fecal impaction in another patient (4%). The mean length of postoperative hospital stays for cohorts 1 and 2 was 1.97 and 1.68 days, respectively, and the costs accrued due to inpatient days were $13,742 and $14,650, respectively. Table 2 further highlights these outcomes.

**Table 2:** Postsurgical analysis for patients based on time spent in recumbent position after TCR The analysis groups comparing against each other for TCR surgery duration, postsurgical outcomes, and costs accrued are included. These outcomes encompass whether the patients had a CSF leak, surgery-related complications (with an independent category for complications specifically due to local infections at the surgical site), the duration of postoperative inpatient stays, and the accrued costs in U.S. dollars as a result ^*^Statistical significance CSF: cerebrospinal fluid; TCR: tethered cord release

Outcome of interest	Cohort 1 (zero days)	Cohort 2 (greater than or equal to one day)	p value
Average length of the procedure (hours)	2.3	2.7	<0.05^*^
CSF leak	2.8%	4%	>0.99
Postsurgical complications	17.1%	12%	0.72
Postsurgical infection	11%	4%	0.58
Postoperative inpatient stay (days)	1.97	1.68	0.62
Costs accrued in U.S. dollars	13,742	14,650	0.79

## Discussion

This retrospective study investigated the effect of the postoperative recumbent position on the incidence of CSF leaks following TCR surgery in a predominantly pediatric population. In addition, the length of the surgical procedure, postsurgical complications, postsurgical infections, postoperative inpatient stay (measured in days), and the cost-effectiveness of time spent in the recumbent position were examined.

Placing the patient in a recumbent position for one to three days postoperatively is a widely utilized approach that is thought to aid in optimizing healing and minimizing the risk of complications, including CSF leaks [9-11]. Our institutional retrospective study revealed that CSF leaks following TCR surgery were observed in 2.8% of patients in cohort 1, who spent zero days in a recumbent position, and 4% in cohort 2, who spent one or more days in this position. In addition, patients in cohort 1 had a lower incidence of CSF leaks by 1.22% compared to cohort 2 following TCR surgery (p > 0.99). Cohort 1 experienced additional postoperative complications besides CSF leak, including areflexia, urinary incontinence, urinary retention, and infection at the incisional site. Additional complications experienced in cohort 2 included fever and fecal impaction.

A prior study found that extending inpatient stays to enforce a flat position after TCR did not reduce the risk of CSF leaks [8]. Univariate analysis demonstrated no significant correlation between complication rates and factors such as operating time, use of dural sealant, microscope use, or the duration of postoperative recumbency. Another study demonstrated no benefit of conservative management protocols (e.g., prone positioning) in over 250 pediatric patients post-TCR [12]. A study by Kanematsu et al. examined the impact of postoperative horizontal positioning in 313 pediatric patients who underwent TCR surgery for tight filum terminale and found no correlation between maintaining patients in a horizontal position and preventing CSF leaks [13]. Additionally, since many TCR patients are young children, staying flat for extended periods presents challenges for patients and their caregivers. The approach of maintaining patients flat after TCR does not appear to lower CSF leak rates. More emphasis should be placed on suturing material and surgical technique rather than recumbent positioning [3,8].

Requiring patients to remain in the hospital after TCR with no inherent therapeutic benefit may significantly increase their medical costs, leading to an unnecessary financial burden on the patient, their families, and the healthcare system. The mean length of postoperative hospital stays for cohorts 1 and 2 was 1.97 and 1.68 days, respectively, and the year-adjusted costs accrued because of inpatient days were $13,742 and $14,605, respectively. This study did not demonstrate a significant difference between cost accrued and inpatient hospital days since cohort 1 had a nonsignificantly higher rate of other complications leading to patients being hospitalized for more extended periods. However, it is not unreasonable to conclude that, when excluding patient complications due to causes other than CSF leaks and requiring patients to spend one to two extra days in the inpatient setting, the cost accrued would be higher for patients required to remain in the recumbent position.

CSF leaks following surgical procedures contribute to substantial additional medical costs. Patients experiencing a postoperative CSF leak need to undergo extended surgical and nonsurgical interventions, requiring prolonged inpatient hospital length of stay and often outpatient management. In addition, CSF leaks can lead to additional complications, including infection and the development of pseudomeningocele. These factors impose a considerable economic burden on the patient and the healthcare system. A study by Charalambous et al. examined the additional costs incurred by patients diagnosed with CSF leaks within 30 days of spinal surgery [14]. They found that the median total cost across groups was $5,101. However, the management of the CSF leaks varied depending on the severity of the patient. Patients who required a lumbar drain accrued an additional $22,341, while those requiring surgical intervention spent an extra $30,199. The cost of managing CSF leaks with epidural blood patch management of CSF was $8,140, while conservative management accounted for an additional $17,012. Another study by Grotenhuis, conducted in the Netherlands, found that postprocedural CSF leaks were associated with 21.7% of the total costs of all spinal procedures and an average of €1508 per patient [15]. While these high costs highlight the importance of identifying CSF leaks postoperatively in various neurosurgical procedures, the data presented in this study suggest that maintaining patients in the recumbent position after cord detethering does not reduce the prevalence of CSF leaks.

Limitations

Our study demonstrated similar rates of CSF leaks between patients who were recumbent for zero days vs. at least one day postoperatively after TCR surgery. Similarly, Chern et al. concluded that extended hospital stays to maintain flat patient positioning after TCR did not prevent CSF leakage [8]. A significant limitation of Chern et al.’s study was the sample size of 222 patients, with which they concluded that a larger sample size would be needed to detect more minor differences in complication rates. Although we could not provide a larger sample size in this study, we hope to increase the evidence in this literature to move the field forward regarding delivering optimal care for patients after TCR. Furthermore, our study expands on prior literature by specifically analyzing costs related to TCR and sectioning of filum terminale in the pediatric population. Another limitation relates to the average age within each cohort, with cohort 1 having an older patient population at 9.14 ± 2.51 years compared to 3.94 ± 2.51 years in cohort 2. This difference could explain some of the differences in outcomes related to surgical complications. Despite these limitations, our study adds to the limited data investigating CSF leaks after TCR.

## Conclusions

This institutional retrospective study examined the impact of postoperative recumbency duration on CSF leak rates following TCR surgery, revealing no significant difference between patients who remained recumbent for at least one day and those who did not remain recumbent. These findings align with prior research suggesting that prolonged recumbency does not reduce CSF leak incidence and may contribute to unnecessary inpatient costs. While the study was limited by its sample size, it provides valuable insights into postoperative management practices and highlights the need for further research to optimize protocols while minimizing patient burden. Future studies should consider larger, multicenter cohorts to better assess potential confounding factors and further validate our findings.
